# Integrative characterization of the heat shock transcription factors in *Acorus*: evolution and heat-responsive regulatory patterns

**DOI:** 10.3389/fpls.2026.1974158

**Published:** 2026-10-07

**Authors:** Liang Ma, Cheng-Yuan Zhou, Yao He, Houhua Fu, Chenyu Gong, Yu Chen, Zhong-Jian Liu, Shipin Chen

**Affiliations:** 1 Fujian Health College, Fuzhou, Fujian, China; 2 College of Forestry, Fujian Agriculture and Forestry University, Fuzhou, China; 3 College of Pharmacy, Fujian University of Traditional Chinese Medicine, Fuzhou, China

**Keywords:** Acorus, co-expression network, comparative genomics, heat stress, HSF gene family, yeast two-hybrid

## Abstract

With global climate change driving rising temperatures, the persistence and physiological performance of *Acorus* are increasingly threatened, highlighting the importance of understanding its molecular responses to heat stress. Heat shock transcription factors (HSFs) constitute a major family of transcriptional regulators involved in plant heat-stress responses and thermotolerance. Here, we identified 39 and 20 HSF genes in *Acorus calamus* and *A. gramineus*. Conserved motif and domain analyses revealed subfamily-specific structural features, while comparative synteny suggested that polyploidy and differential gene retention contributed to HSF family expansion in *A. calamus*. Subgenome-level analysis revealed no obvious difference in HSF gene number between subgenomes, whereas retained homeolog pairs exhibited tissue-dependent expression divergence. Promoter, predicted protein–protein interaction, and GO enrichment analyses linked AgHSFs to stress regulation and heat-response networks. Integrated analysis of time-series expression profiles, differential expression, weighted gene co-expression networks, and qRT-PCR revealed diverse temporal responses among AgHSFs and supported AgHSF07, AgHSF12, and AgHSF20 as strongly heat-responsive candidates for further characterization. AgHSF07 and AgHSF12 showed mainly nuclear YFP signals and strong autoactivation in the yeast system, supporting their potential roles as transcriptional activators. Together, this integrated genomic–transcriptomic framework provides insights into HSF evolution and heat-response regulation in *Acorus* and highlights AgHSF07 and AgHSF12 as promising candidate regulators for further functional studies of thermotolerance.

## 1 Introduction

Heat stress has become an increasingly serious challenge for plants under a warming climate, as increasing average temperatures and more frequent heatwaves are affecting both natural ecosystems and agricultural production worldwide (Hatfield and Prueger, 2015). When temperatures exceed the optimal range for growth, plants commonly show reduced photosynthetic efficiency, impaired water relations, and altered carbon allocation, which together restrict vegetative development and biomass accumulation (Zinn et al., 2010; Mahajan et al., 2025). Plants have therefore evolved complex physiological and molecular mechanisms to perceive elevated temperatures and activate protective responses that maintain cellular stability and promote survival under thermal stress (Wang et al., 2024; Mircea et al., 2025). As climate warming continues to intensify, understanding the regulatory mechanisms of plant heat responses has become essential for maintaining growth performance, preserving ornamental value, and supporting the breeding of heat-resilient plants.

Heat shock transcription factors (HSFs) are conserved transcriptional regulators with central roles in plant heat-stress responses (Scharf et al., 2012). HSFs recognize heat shock elements located in target-gene promoters and activate transcriptional programs involved in protein protection, cellular homeostasis, and adaptation to elevated temperatures (Guo et al., 2016). Plant HSF proteins typically possess a conserved DNA-binding domain (DBD) followed by an oligomerization domain. Based on their structural features, HSFs are grouped into three classes, HSFA, HSFB, and HSFC, which represent distinct evolutionary and functional lineages within the family (Nover et al., 2001; Guo et al., 2016). Comparative analyses across diverse lineages have shown that HSF genes are highly conserved but have also diversified during plant adaptation to changing thermal environments (Yu et al., 2022). Recent studies have further revealed the roles of specific HSF members in heat tolerance, supporting their value as targets for improving plant performance under heat stress (Kang et al., 2025; Zhu et al., 2025; Xu et al., 2026).

The genus *Acorus* is a distinctive monocot lineage of broad biological significance and long-standing research interest, with considerable horticultural and ornamental value (Shu et al., 2018; Cheng et al., 2020). *Acorus* also has ecological importance in wetland systems and a long history of medicinal and ethnobotanical use (Zhang et al., 2016; Zhao et al., 2023). However, under rapid global climate change, *Acorus* species growing in freshwater wetlands and humid habitats are likely to face increasing challenges to growth and survival (Yue et al., 2024). Despite the central role of HSFs in plant thermal responses (Yu et al., 2022), this family has not been systematically examined in *Acorus*. The limited knowledge of HSF-mediated heat responses in *Acorus* hinders the identification of key heat-responsive regulators and their potential use in breeding. Systematic analysis of HSFs will improve our understanding of heat-response regulation and facilitate genetic improvement for heat tolerance in *Acorus* germplasm.

Given the key functions of HSFs in plant heat stress responses and the increasing thermal challenges associated with climate warming, a systematic analysis of HSFs was conducted in *A. calamus* and *A. gramineus*. The evolutionary relationships and genomic features of *Acorus* HSFs were investigated through phylogenetic, conserved motif, chromosomal distribution, synteny, and cis-regulatory element analyses. Heat-responsive AgHSFs were further examined by integrating time-series transcriptome data, differential expression results, weighted gene co-expression networks, and qRT-PCR validation. Selected candidate genes were subsequently characterized by subcellular localization and yeast two-hybrid assays to investigate their cellular localization and regulatory properties. These findings improve our understanding of HSF evolution and heat-responsive regulation in *Acorus* and provide a basis for the genetic improvement and utilization of heat-tolerant germplasm.

## 2 Materials and methods

### 2.1 HSF identification and physicochemical profiling in *Acorus*

Genome assemblies and annotations for *A. calamus* and *A. gramineus* were obtained from NCBI (Ma et al., 2023). The longest transcript was retained to generate the representative protein dataset. *Arabidopsis thaliana* HSF sequences were retrieved from PlantTFDB (Jin et al., 2014).

HSF candidates were identified using both HMM- and BLASTP-based searches. The HMM profile of the HSF DNA-binding domain (PF00447.24) was downloaded from Pfam (Mistry et al., 2021) and searched against the representative protein datasets with HMMER v3.3.2 (Potter et al., 2018). The Pfam gathering threshold was applied using the --cut_ga option, corresponding to sequence and domain scores of 22 bits for PF00447. In parallel, *Arabidopsis* HSF proteins served as BLASTP queries against the two *Acorus* protein datasets using an E-value threshold of 1 × 10^-5^ (Camacho et al., 2009). Candidates identified by either HMMER or BLASTP were combined and subjected to conserved-domain verification.

Physicochemical characteristics of HSF proteins were determined with ExPASy ProtParam (Gasteiger et al., 2005).

### 2.2 Phylogenetic analysis of AcHSFs and AgHSFs

HSF protein sequences were aligned using MAFFT (Katoh and Standley, 2013). An ML phylogenetic tree was conducted in IQ-TREE (Nguyen et al., 2015). ModelFinder selected JTT+R4 as the best-fitting substitution model according to the Bayesian Information Criterion (Kalyaanamoorthy et al., 2017). The tree was annotated in iTOL (Letunic and Bork, 2024). AcHSFs and AgHSFs were classified into the HSFA, HSFB, and HSFC groups based on their relationships with *Arabidopsis* HSF proteins.

### 2.3 Conserved motif and structural feature analysis of AcHSFs and AgHSFs

Conserved motifs and canonical structural features of AcHSF and AgHSF proteins were analyzed using complementary sequence-based methods. Conserved motifs were identified with MEME Suite (Bailey et al., 2015), with the maximum number of motifs set to 10 and other parameters kept at default settings. The DNA-binding domain (DBD) was defined based on PF00447 HMMER searches and NCBI Conserved Domain Database annotation (Lu et al., 2020). The HR-A/B oligomerization domain was predicted using MARCOIL with a coiled-coil probability threshold of 50% (Delorenzi and Speed, 2002). HSF subclass assignments and HR-A/B organization were further checked by MAFFT (Katoh and Standley, 2013) alignment with representative *Arabidopsis thaliana* HSFs and reference to the HEATSTER classification framework (Berz et al., 2019).

Nuclear localization signals (NLSs) were predicted using NLStradamus with a threshold of 0.6 (Nguyen Ba et al., 2009), whereas nuclear export signal (NES) candidates were predicted using NESmapper v1.1 with the activity-based profile and a minimum score of 2 (Kosugi et al., 2014). AHA activation motifs were examined in HSFA proteins by aligning *Acorus* HSFAs with corresponding *A. thaliana* HSFs and comparing homologous regions with previously characterized AHA motifs (Döring et al., 2000; Kotak et al., 2004; Lin et al., 2014). Conserved motifs and DBD organization were visualized using TBtools-II (Chen et al., 2023).

### 2.4 Chromosomal distribution and collinearity analysis

The chromosomal positions of AcHSF and AgHSF genes were visualized with TBtools-II (Chen et al., 2023). Linkage-group lengths were obtained with SAMtools (Li et al., 2009). Gene density was calculated using BEDTools (Quinlan and Hall, 2010). To identify duplicated gene pairs and collinear blocks within each genome, self-comparison analysis was performed using MCScanX (Wang et al., 2012). The final circle plot was generated using Circos (Krzywinski et al., 2009). The interspecific synteny analysis was performed using the JCVI package (Tang et al., 2024).

### 2.5 Subgenome distribution and homeolog retention analysis of AcHSFs

AcHSFs were assigned to the A or B subgenome according to their genomic positions and the subgenome assignments of *A. calamus* (Ma et al., 2023). A/B homeologous HSF pairs were identified from the published *A. calamus* homeolog dataset (Ma et al., 2023). Relationships between AcHSFs and AgHSFs were determined mainly by interspecific synteny, with BLASTP similarity and phylogenetic relationships used to resolve ambiguous cases (Cheng et al., 2012; Zhao et al., 2021a; Walden and Schranz, 2023).

Expression divergence between retained AcHSF homeologs was assessed using the seven-tissue expression dataset of *A. calamus* (Ma et al., 2023). Following Zhang et al. (2021), homeolog expression bias (HEB) was calculated as the log2 expression ratio of the A- to B-subgenome copies. When either expression value of a homeolog pair was <1, a pseudocount of 1 was added to both values before calculating the ratio. Positive and negative HEB values indicate higher expression of the A- and B-subgenome copies, respectively. Values with |HEB| > 1 were classified as descriptively biased.

### 2.6 Cis-regulatory element analysis

For each HSF gene, the 2-kb upstream sequence was obtained from the genomes of the two *Acorus* species. Cis-regulatory elements were predicted with PlantCARE (Lescot et al., 2002) and assigned to four functional categories related to plant growth, phytohormone response, light response, and abiotic stress. The abundance and composition in AcHSF and AgHSF promoters were visualized with ggplot2 (Wickham, 2016).

### 2.7 Protein interaction network and GO enrichment analysis of AgHSFs

AgHSF proteins were submitted to STRING (Szklarczyk et al., 2023), with *Arabidopsis thaliana* specified as the reference organism. AgHSF proteins were mapped to their corresponding *Arabidopsis* homologs, and a predicted protein association network was generated based on STRING annotations. The predicted network was then transferred to Cytoscape for visualization and layout optimization (Shannon et al., 2003).

Functional enrichment of AgHSFs was assessed by Gene Ontology (GO) analysis. The identified AgHSFs were defined as the foreground set, whereas GO-annotated proteins from the *A. gramineus* proteome served as the reference background. Protein GO annotations were generated with eggNOG-mapper (Cantalapiedra et al., 2021), and GO relationships were defined according to the ‘go-basic.obo’ ontology file from the Gene Ontology resource (The Gene Ontology Consortium, 2019). Enrichment was tested using a hypergeometric test, followed by Benjamini–Hochberg correction across all tested terms. Terms with a BH-adjusted P < 0.05 were regarded as significant. Enriched GO terms were analyzed and plotted with clusterProfiler in R (Yu et al., 2012).

### 2.8 Plant materials and heat stress treatment

Rhizome divisions were obtained from healthy, five-year-old *A. gramineus* plants grown in the Fujian Health College germplasm nursery and propagated asexually. After six months of greenhouse cultivation, uniform plants with at least six intact leaves, a height of approximately 30 cm, healthy root systems, and no visible disease symptoms were chosen for the experiment. Before heat treatment, the selected plants were maintained under controlled conditions for 10 days, with a 12 h light/12 h dark photoperiod, 8, 000 lx light intensity, day/night temperatures of 28/25 °C, and 75% relative humidity.

Plants were then exposed to 40 °C for heat treatment. Leaves collected before treatment were used as the 0 h control, and subsequent samples were taken at 2, 6, 12, 24, 48, and 72 h. Each time point included three biological replicates. After collection, the leaves were immediately placed in liquid nitrogen and then kept at −80 °C.

### 2.9 Transcriptome-based expression profiling and differential expression analysis of AgHSFs under heat stress

Total RNA from *A. gramineus* leaves was isolated with the FastPure Plant Total RNA Isolation Kit following the manufacturer’s protocol. RNA-seq libraries were then prepared and sequenced on an Illumina NovaSeq 6000 platform. Raw transcriptome reads were filtered by fastp (Chen et al., 2018), followed by mapping to the *A. gramineus* reference genome with HISAT2 (Kim et al., 2019). Gene-level read counts were obtained with featureCounts (Liao et al., 2014). AgHSF expression profiles were visualized using the R package pheatmap (Kolde, 2025).

Differential expression at each heat-treatment time point relative to the 0 h control was assessed with DESeq2 (Love et al., 2014). Genes meeting both an adjusted P value < 0.01 and an absolute log_2_ fold change (|log_2_FC|) ≥ 2 were defined as differentially expressed under the stringent criterion used in this study. For visualization of AgHSF expression patterns, DESeq2 size-factor-normalized counts were log_2_-transformed after addition of a pseudocount and subsequently standardized within each gene (Z-score). AgHSF genes lacking complete and reliable differential-expression estimates because of very low transcript abundance were excluded from the temporal differential-expression visualization.

### 2.10 Gene co-expression network and module analysis

Gene co-expression patterns were examined with the WGCNA package in R (Langfelder and Horvath, 2008). Genes showing CPM ≥ 1 in at least three samples were kept, and count data were variance-stabilized using DESeq2 (blind = TRUE). A signed network based on biweight midcorrelation (bicor) was built with a soft-thresholding power of 18. Power 18 was selected with reference to previous plant WGCNA studies using an R² of 0.75 (Yang et al., 2025; Zhou et al., 2026). Network modules were detected with blockwiseModules using minModuleSize = 50, deepSplit = 2, mergeCutHeight = 0.30, and maxBlockSize = 6000.

Module eigengenes were used to evaluate temporal associations with heat treatment. To evaluate module-level temporal responses, module eigengenes were compared across the seven time points using one-way ANOVA, with time treated as a categorical factor. The obtained P values were subjected to multiple-testing correction by the Benjamini–Hochberg procedure (Benjamini and Hochberg, 1995), and modules showing FDR < 0.05 were defined as temporally responsive. Module membership (kME) was calculated by bicor, and AgHSF genes with kME ≥ 0.80 were considered to have high intramodular membership.

### 2.11 Quantitative real-time PCR validation

AgHSF genes were chosen for qRT-PCR validation based on combined evidence from heat-responsive expression patterns, strict differential-expression analysis, and WGCNA. Additional leaf samples were independently collected, and total RNA was separately extracted and used for first-strand cDNA synthesis with the Hifair AdvancedFast One-Step RT-gDNA Digestion SuperMix for qPCR (Yeasen, Shanghai, China). Primer pairs were designed in Primer Premier 5.0 (Lalitha, 2000) and are listed in **Supplementary Table S1**. qRT-PCR was conducted on a QuantStudio 1 Plus Real-Time PCR Detection System with Hieff UNICON Universal Blue qPCR SYBR Green Master Mix (Yeasen, Shanghai, China). Thermal cycling consisted of 30 s at 95 °C, followed by 40 cycles of 15 s at 95 °C and 30 s at 60 °C. DESeq2-derived log_2_ fold changes were compared with qRT-PCR log_2_ fold changes calculated as −ΔΔCt relative to 0 h. Pearson and Spearman correlations were calculated across 66 matched gene–time observations, and a least-squares regression line was fitted for visualization.

*AgACT1* (KAK1276548.1) served as the internal reference gene, following the reference gene selection strategy in previous study (Ma et al., 2025). The analysis comprised three independent biological replicates, each measured in triplicate. Relative expression was determined using the 2^−ΔΔCt^ method (Livak and Schmittgen, 2001).

### 2.12 Subcellular localization

Full-length AgHSF07, AgHSF12, and AgHSF20 coding sequences were recombined into pEarleyGate104 to produce N-terminal YFP fusions (Earley et al., 2006) and introduced into *Agrobacterium tumefaciens* GV3101. Cultures containing the fusion constructs (OD_600_ = 0.5) were mixed with P19 (OD_600_ = 0.3) in infiltration buffer containing 10 mM MES, 10 mM MgCl_2_, and 100 μM acetosyringone and incubated at room temperature in the dark for at least 3 h. The resulting suspensions were introduced into leaves of 3–5-week-old *Nicotiana benthamiana* according to the method of Sparkes et al. (2006), with slight modifications. After infiltration, the plants were maintained in darkness for 24 h and then exposed to normal light for 48 h. YFP and RFP fluorescence signals were visualized using a ZEISS LSM 900 confocal laser-scanning microscope (Carl Zeiss Microscopy GmbH, Jena, Germany). Empty pEarleyGate104 and the corresponding subcellular markers were included as controls.

### 2.13 Transcriptional activation activity and yeast two-hybrid analysis

The transcriptional activation activity of AgHSF07, AgHSF12, and AgHSF20 was first assessed using the GAL4-based yeast system. The coding sequences of *AgHSF07*, *AgHSF12*, and *AgHSF20* were cloned into pGBKT7, and each BD fusion construct was co-transformed with empty pGADT7 to test for transcriptional activation activity.

Candidate interaction partners were subsequently selected from the STRING-predicted protein association network. Sequence similarity and expression-profile concordance with the corresponding AgHSFs were used as additional criteria to prioritize candidate partners, because integrating co-expression information with predicted or experimentally supported protein association networks can provide additional biological context for candidate selection (Ding and Kihara, 2019; Piya et al., 2014). The coding sequences of the selected candidate partners were cloned into pGBKT7, and their autoactivation activity was examined by co-transformation with empty pGADT7. Because BD-AgHSF07 and BD-AgHSF12 showed autoactivation, pairwise interaction assays were performed with AgHSF07, AgHSF12, and AgHSF20 fused to the GAL4 activation domain in pGADT7 and their corresponding candidate partners fused to the GAL4 DNA-binding domain in pGBKT7.

The pGBKT7-53/pGADT7-T and pGBKT7-Lam/pGADT7-T combinations were used as positive and negative controls, respectively. Plasmid pairs were introduced into Y2HGold cells using the LiAc/heat-denatured salmon sperm carrier DNA/PEG method (Gietz and Schiestl, 2007). Transformants grown on SD/−Trp/−Leu medium were adjusted to comparable cell densities, serially diluted tenfold, and spotted onto SD/−Trp/−Leu, SD/−Trp/−Leu/−His/−Ade, and SD/−Trp/−Leu/−His/−Ade/X-α-Gal media. Plates were incubated at 30 °C.

To examine potential HSF recognition sites in the candidate partner genes, the 2-kb upstream sequences were obtained. Canonical HSEs were identified by searching for the consensus sequences 5′-NGAANNTTCNNGAAN-3′ and 5′-NTTCNNGAANNTTCN-3′, each comprising three contiguous pentameric units arranged in alternating orientations, where N denotes any nucleotide (Amin et al., 1988; Zhao et al., 2020).

## 3 Results

### 3.1 Identification and characterization of HSF genes in *Acorus*

A total of 39 and 20 HSF genes were identified in *A. calamus* and *A. gramineus*, respectively. These genes were designated *AcHSF01–AcHSF39* and *AgHSF01–AgHSF20*, respectively. Detailed information for AgHSFs and AcHSFs is listed in **Supplementary Table S2**.

The AcHSF proteins comprised 181–562 amino acids (AcHSF17 and AcHSF04, respectively) and had predicted masses of 21.19 kDa (AcHSF17) to 62.19 kDa (AcHSF04) (**Supplementary Table S3**). Predicted pI values for AcHSFs extended from 4.67 for AcHSF04 to 10.30 for AcHSF12. The instability index ranged from 37.60 (AcHSF15) to 75.84 (AcHSF17), the aliphatic index ranged from 55.75 (AcHSF10 and AcHSF26) to 88.57 (AcHSF19), and the GRAVY values ranged from −0.820 (AcHSF35) to −0.421 (AcHSF19). The AgHSF proteins comprised 167–497 amino acids (AgHSF17 and AgHSF07, respectively) and had predicted masses of 19.58 kDa (AgHSF17) to 55.44 kDa (AgHSF07). Their theoretical pI values extended from 4.76 for AgHSF09 to 9.06 for AgHSF15. The instability index ranged from 40.09 (AgHSF03) to 69.65 (AgHSF08), the aliphatic index ranged from 55.16 (AgHSF13) to 84.10 (AgHSF07), and GRAVY values ranged from −0.812 (AgHSF06) to −0.401 (AgHSF07).

### 3.2 Phylogenetic relationships of AcHSFs and AgHSFs

Phylogenetic analysis of 39 AcHSFs, 20 AgHSFs, and 21 AtHSFs revealed the relationships among HSF members from the three species (**Figure 1**). Among the three major groups, HSFA contained the largest number of AcHSF and AgHSF members, while HSFC contained the fewest. In the phylogenetic tree, AcHSFs and AgHSFs were distributed together within the three major groups rather than forming completely separate species-specific branches. The overall distribution pattern showed that HSF proteins from the two *Acorus* species were mainly concentrated in HSFA and HSFB, with only a small number of members present in HSFC.

**Figure 1:**
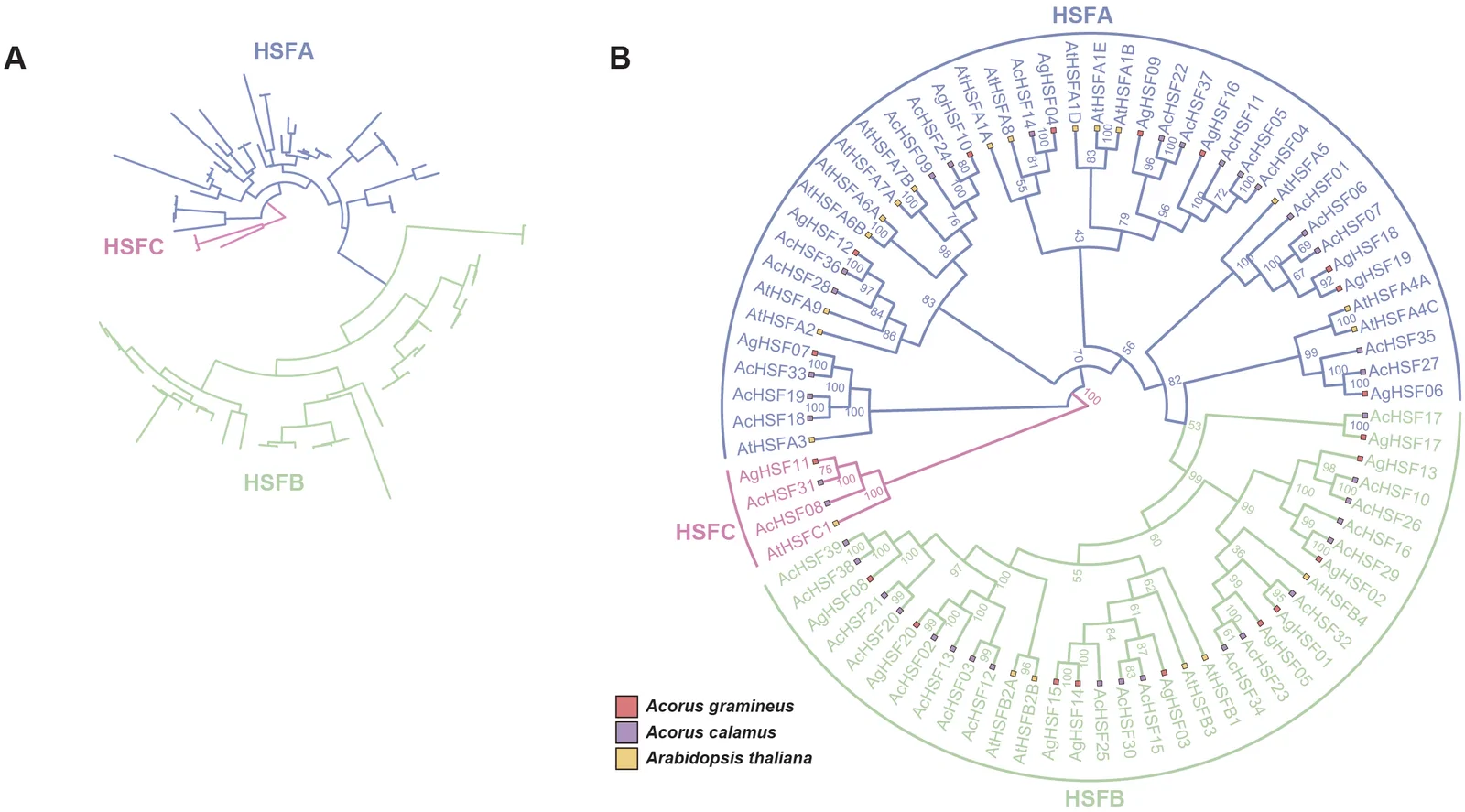
Phylogenetic relationships and subfamily classification of HSF proteins in *A. calamus*, *A. gramineus*, and *Ara. thaliana*. **(A)** Phylogenetic topology; **(B)** Maximum-likelihood phylogenetic tree of HSF proteins.

### 3.3 Conserved motifs and structural features of AcHSFs and AgHSFs

The conserved motif composition of AcHSFs and AgHSFs was analyzed using 10 predicted motifs (**Figure 2**). All AcHSF and AgHSF proteins contained motif 1 (**Figure 2B**). In contrast, the distributions of the other motifs varied among different proteins and phylogenetic branches.

**Figure 2:**
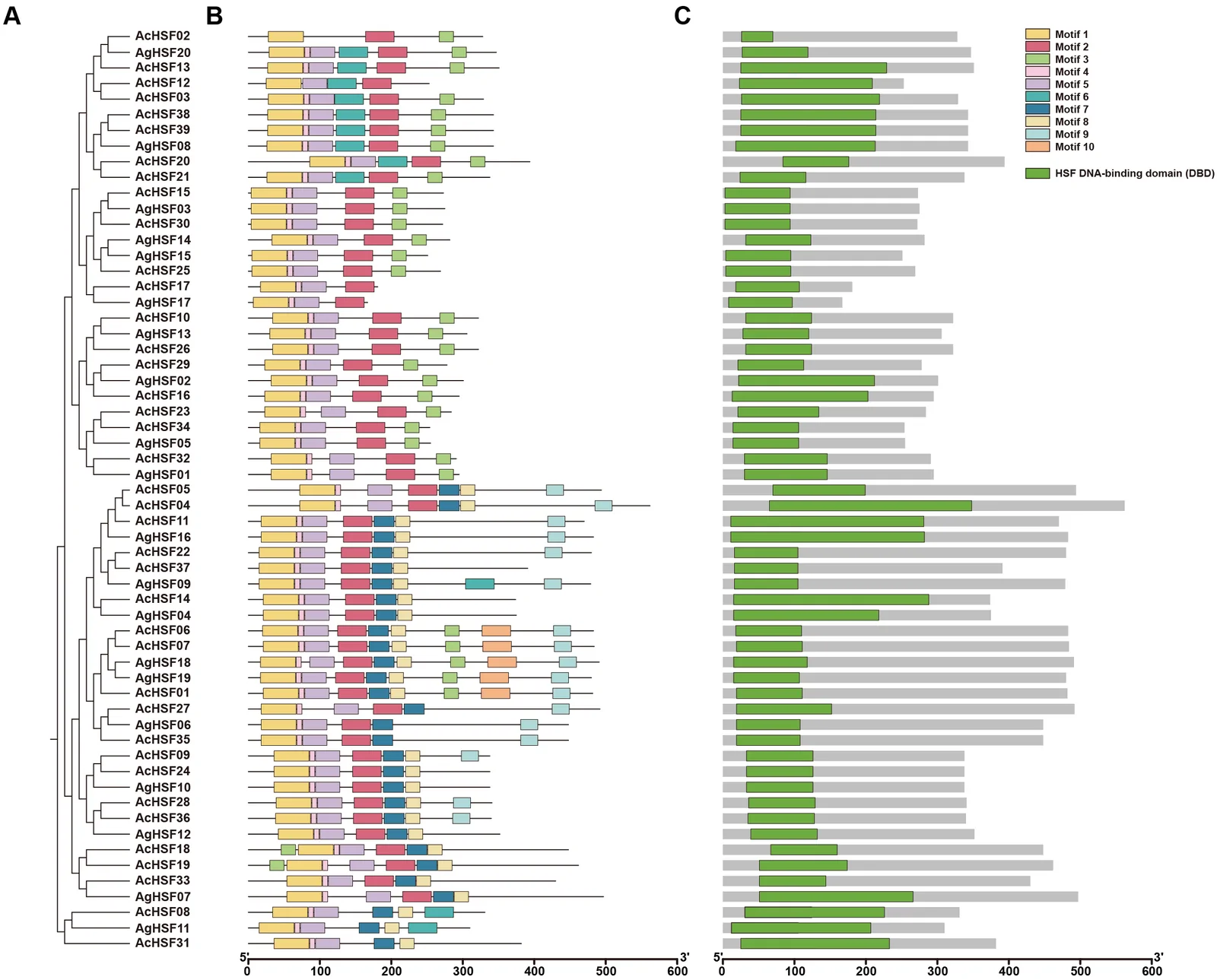
Phylogenetic relationships and conserved structural features of *Acorus* HSF proteins. **(A)** Phylogenetic tree containing AcHSF and AgHSF proteins; **(B)** Conserved-motif composition; **(C)** Distribution and positions of the conserved HSF DNA-binding domains (DBD). The legends for motifs and domains are provided on the right.

The motif arrangement was generally consistent with the clustering pattern shown in **Figure 2A**. Proteins that were closely clustered in the phylogenetic tree usually showed similar motif composition and order. Clear differences in motif composition were observed among the three HSF subfamilies. The HSFC group, including AcHSF08, AgHSF11, and AcHSF31, showed highly similar motif arrangements and contained fewer motif types than most HSFA and HSFB members. By contrast, both HSFA and HSFB proteins displayed more diverse motif combinations. The conserved HSF domain was present in all AcHSF and AgHSF proteins (**Figure 2C**).

The canonical structural features of the 59 *Acorus* HSF proteins were further analyzed (**Figure 2**; **Supplementary Table S4**). All AcHSF and AgHSF proteins contained a conserved DNA-binding domain (DBD) and an HR-A/B oligomerization region, although the positions and lengths of the HR-A/B regions varied among different HSF members. Nuclear localization signals (NLSs) were predicted in 17 proteins, including 10 HSFA and seven HSFB members, whereas no NLS was detected in the three HSFC proteins. Predicted nuclear export signal (NES) candidates were identified in 21 proteins, including 20 HSFA members and one HSFC member, whereas no NES candidate was detected in HSFB proteins.

AHA motifs were further examined in HSFA proteins. Recognizable AHA-type motifs were identified in 25 of the 29 HSFA members. The A2 proteins contained two AHA-like motifs, while the A3 proteins showed a conserved W-X16-W-X15-W pattern. Conserved AHA-type sequences were also identified in the A1, A4, A5, and A6 subclasses. No confidently assignable AHA motif was identified in AcHSF14, AcHSF25, AgHSF04, or AgHSF15.

### 3.4 Chromosomal distribution, duplication patterns, and synteny

Genome-wide collinearity analysis revealed extensive syntenic relationships among different chromosomal regions of *A. gramineus* (**Figure 3A**). Within the AgHSF genes, only a limited number of syntenic gene pairs were identified, and these collinear connections were mainly detected between different linkage groups.

**Figure 3:**
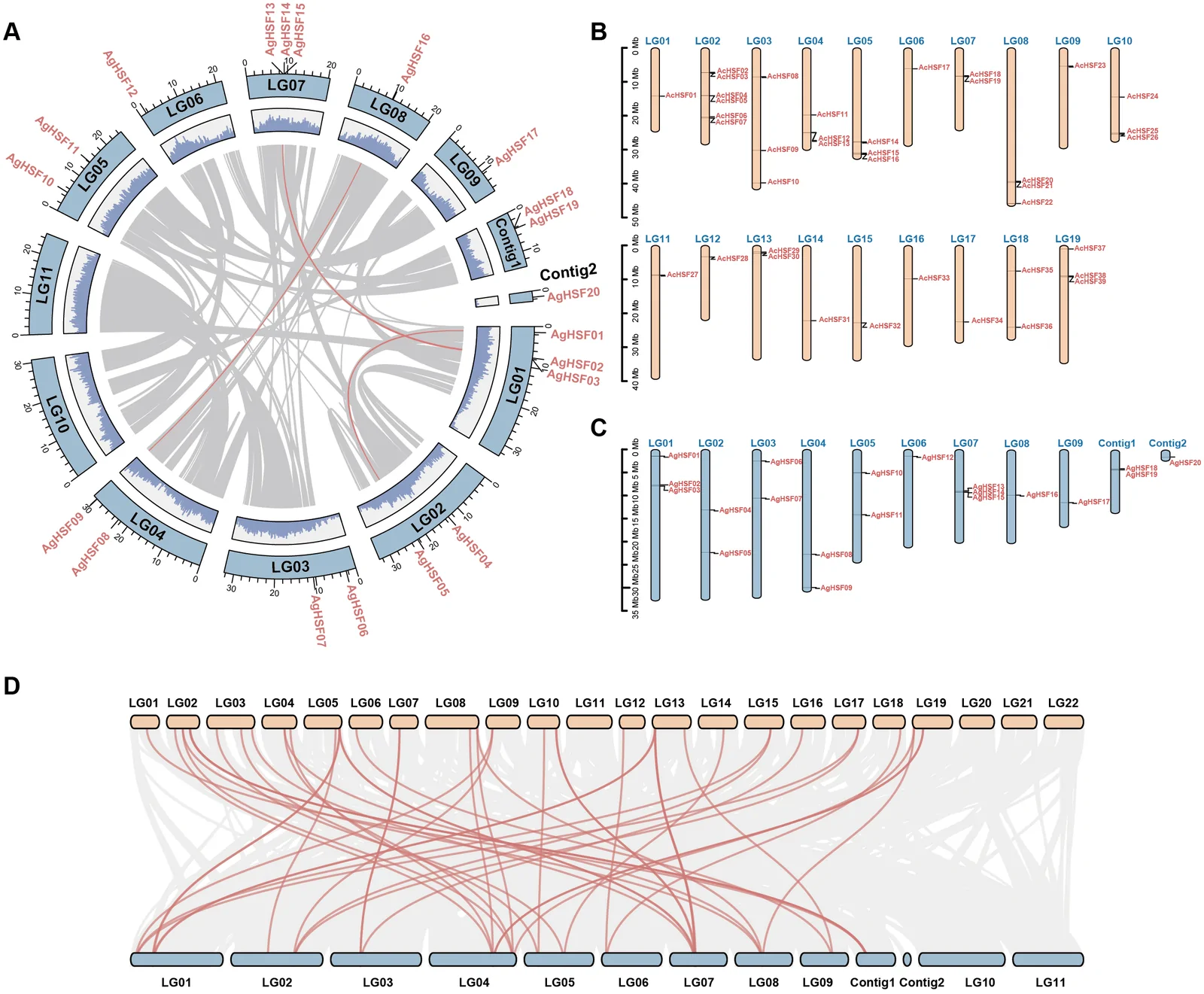
Chromosomal distribution and synteny analysis of HSF genes in *A. calamus* and *A. gramineus*. **(A)** Chromosomal distribution and intraspecific collinearity of AgHSFs; **(B)** Chromosomal localization of AcHSFs; **(C)** Chromosomal localization of AgHSFs; **(D)** Interspecific syntenic relationships of AcHSFs and AgHSFs.

Subsequently, we investigated the chromosomal distribution patterns of HSF genes in the two species. In *A. calamus*, HSF genes showed an uneven distribution and local clustering. The clearest clusters occurred on LG02 (*AcHSF02*–*AcHSF07*), LG07 (*AcHSF13*–*AcHSF16*), LG10 (*AcHSF24*–*AcHSF26*), and LG19 (*AcHSF37*–*AcHSF39*) (**Figure 3B**). In *A. gramineus*, the 20 AgHSFs were distributed across 11 linkage groups and 2 contigs, with obvious clustering on only a few chromosomes (**Figure 3C**). The main concentration regions were LG01 (*AgHSF01*–*AgHSF03*), LG03 (*AgHSF06*–*AgHSF08*), and LG07 (*AgHSF13*–*AgHSF15*), whereas most of the remaining linkage groups carried only one or two HSF genes.

To further examine the genomic relationships of the HSF gene family between the two species, synteny analysis was performed between *A. calamus* and *A. gramineus* (**Figure 3D**). Widespread collinearity was observed between AcHSF and AgHSF genes, with 15 of the 20 AgHSFs having at least one collinear AcHSF counterpart (**Supplementary Table S5**). Among these, two AgHSFs showed a 1:1 correspondence pattern, three showed 1:2, five showed 1:3, three showed 1:4, and two showed 1:5, whereas five AgHSFs had no detected collinear AcHSF counterpart. These relationships were distributed across different linkage groups rather than being restricted to a single chromosome pair. Among the 39 AcHSFs, 33 were classified as WGD/segmental duplicates and six as dispersed duplicates, indicating that large-scale duplication was the predominant source of AcHSF copy retention.

### 3.5 Subgenome distribution, homeolog retention, and expression divergence of AcHSFs

The AcHSFs showed a nearly balanced distribution between the two subgenomes, with 19 genes assigned to the A subgenome and 20 to the B subgenome (**Supplementary Tables S6**, **S7**). HSFA contained 10 A-subgenome and eight B-subgenome members, HSFB contained eight and 11 members, respectively, and HSFC contained one member in each subgenome, showing no obvious subgenome bias in HSF copy number. Fourteen A/B homeologous pairs, comprising 28 AcHSFs, were identified from the published *A. calamus* homeolog dataset. The unpaired genes included six HSFA and five HSFB members. No pronounced difference was observed between HSFA and HSFB, while HSFC showed complete pair retention in this small subclass.

Homeolog expression bias analysis showed that 10 of the 14 retained AcHSF pairs had |HEB| > 1 in at least one tissue (**Supplementary Figure S1**; **Supplementary Table S8**). Among the 98 pair-by-tissue comparisons, 10 were A-biased and 20 were B-biased. Distinct tissue-dependent bias patterns were observed among different homeolog pairs. AcHSF02/AcHSF13 showed B-biased expression in inflorescence, peduncle, leaf, root, and stem, while AcHSF10/AcHSF26 showed A-biased expression in flower and bract. AcHSF30/AcHSF15 displayed a shift in bias direction, with B-biased expression in flower and bract and A-biased expression in inflorescence, peduncle, and leaf. These results indicate substantial tissue-dependent expression divergence among a subset of retained AcHSF homeologs.

### 3.6 Cis-regulatory elements of *Acorus* HSF promoters

Analysis of HSF promoter regions in *A. calamus* and *A. gramineus* revealed a diverse set of cis-regulatory elements (**Figure 4**). These elements were assigned to four functional groups associated with plant growth, phytohormone response, light response, and abiotic stress. Light-related elements were particularly abundant in both AcHSF and AgHSF promoters and represented the predominant category in most genes. Hormone-related elements were also common, whereas elements associated with plant growth and abiotic stress occurred at lower frequencies. Box 4, G-box, GATA-motif, GT1-motif, and TCT-motif were common components of the light-response group. Among hormone-related elements, ABRE occurred in numerous HSF promoters. Stress-associated motifs included ARE, MBS, LTR, TC-rich repeats, GC-motif, and WUN-motif. Growth-related motifs, including CAT-box, O2-site, MSA-like, GCN4_motif, and HD-Zip 1, were found in a subset of genes.

**Figure 4:**
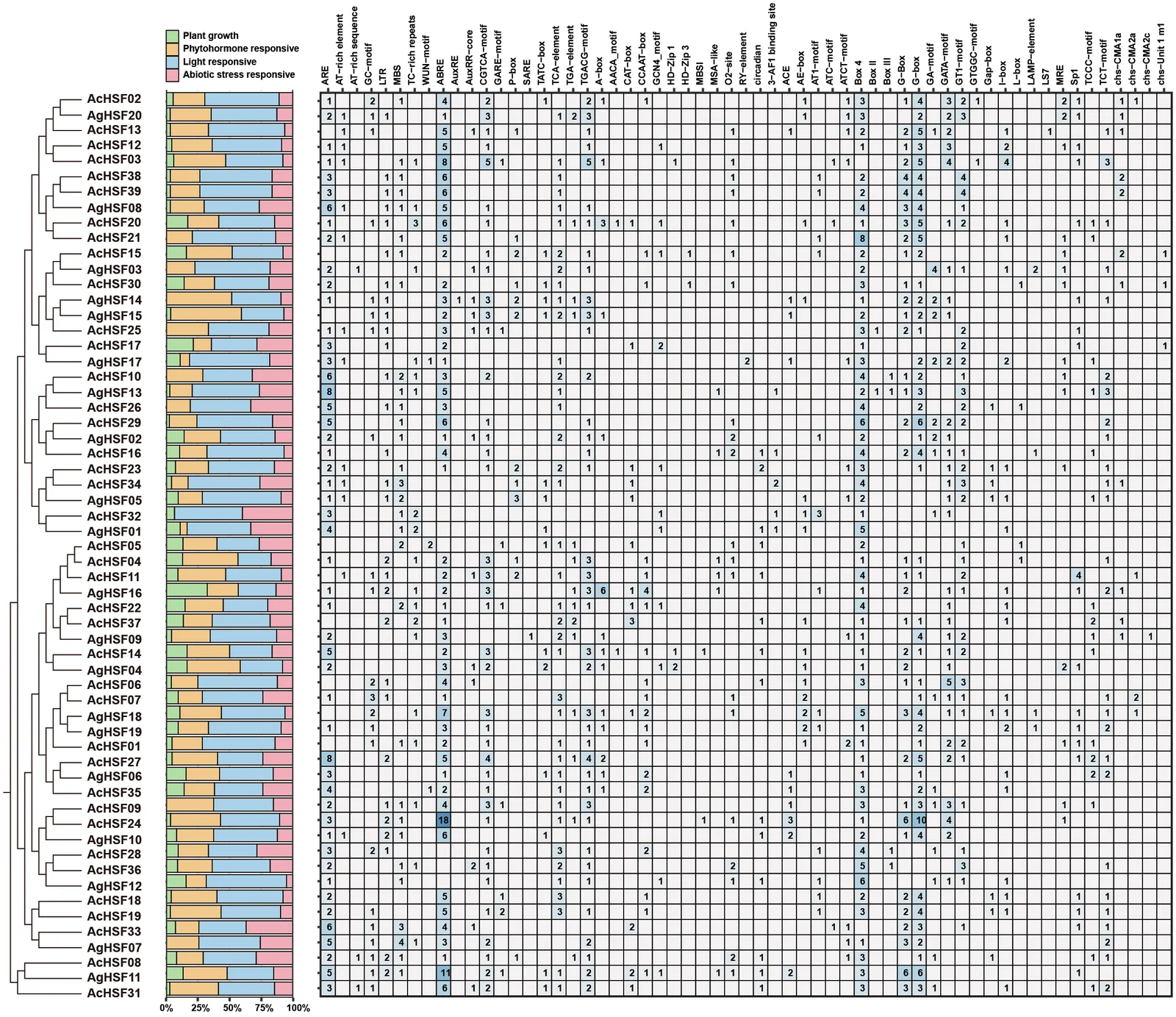
Composition of cis-acting elements in AcHSF and AgHSF promoter*s*.

### 3.7 Protein association network prediction and GO enrichment analysis of AgHSFs

A protein association network was predicted for AgHSF proteins (**Figure 5A**). The AgHSF-associated proteins formed a single network with extensive links to heat stress-related proteins (**Supplementary Table S9**). Most associated nodes belonged to the heat shock protein (HSP) family. Other stress-associated proteins included APX1, APX2, BAG6, CLPB1, DREB2A, FKBP62, GOLS1, HSA32, HSBP, HTT1, HTT2, MBF1C, and SPH25. Several *Arabidopsis* HSF-related proteins, including HSFA1D, HSFA1E, HSFA7A, HSFA7B, HSFA9, and HSFB2B, were also present in the predicted network.

**Figure 5:**
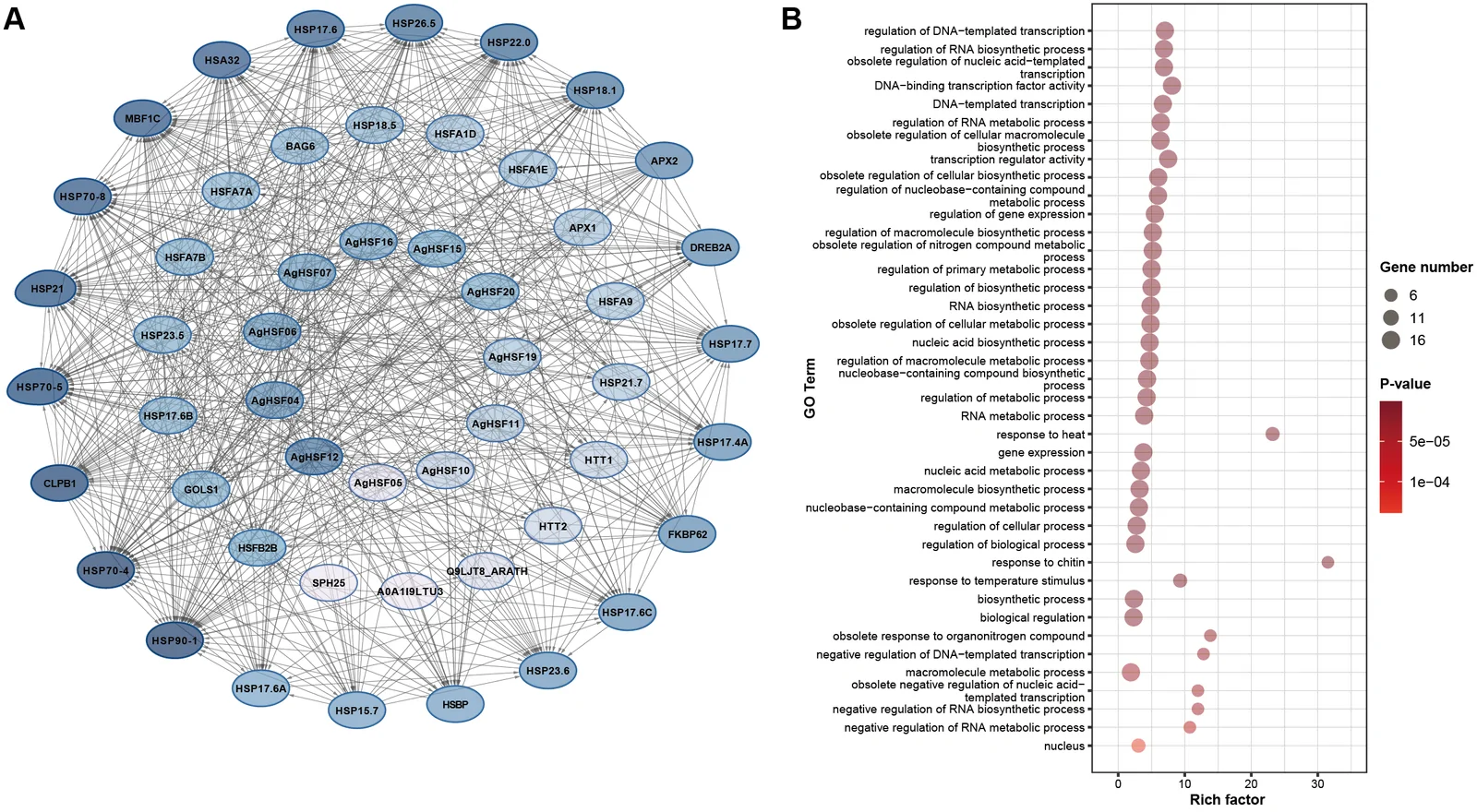
Predicted protein association network and GO enrichment analysis of AgHSFs. **(A)** Predicted protein-protein interaction network of AgHSF-associated proteins; **(B)** GO enrichment analysis of AgHSFs.

GO analysis showed that the main enriched molecular function terms for AgHSFs were transcription regulator activity and DNA-binding transcription factor activity (**Figure 5B**). For biological process, the strongest enrichment was observed for regulation of DNA-templated transcription, RNA biosynthesis, DNA-templated transcription, and RNA metabolism. GO terms containing relatively large numbers of AgHSF genes were mainly related to heat and temperature responses, gene expression, nucleic acid and RNA metabolism, macromolecule biosynthesis, and regulation of biological processes. In addition, enrichment was also observed for response to chitin and response to organonitrogen compound.

### 3.8 Transcriptome-based expression profiling of AgHSFs under heat stress

Transcriptome analysis revealed distinct temporal expression patterns among the 20 AgHSF genes during heat treatment (**Figure 6A**; **Supplementary Table S10**). Several AgHSFs showed pronounced changes during the treatment period. *AgHSF07*, *AgHSF10*, *AgHSF12*, *AgHSF16*, and *AgHSF20* responded rapidly during the early stages of heat treatment, whereas *AgHSF04* and *AgHSF06* showed more evident increases at later time points. In contrast, several other family members exhibited relatively limited temporal variation.

**Figure 6:**
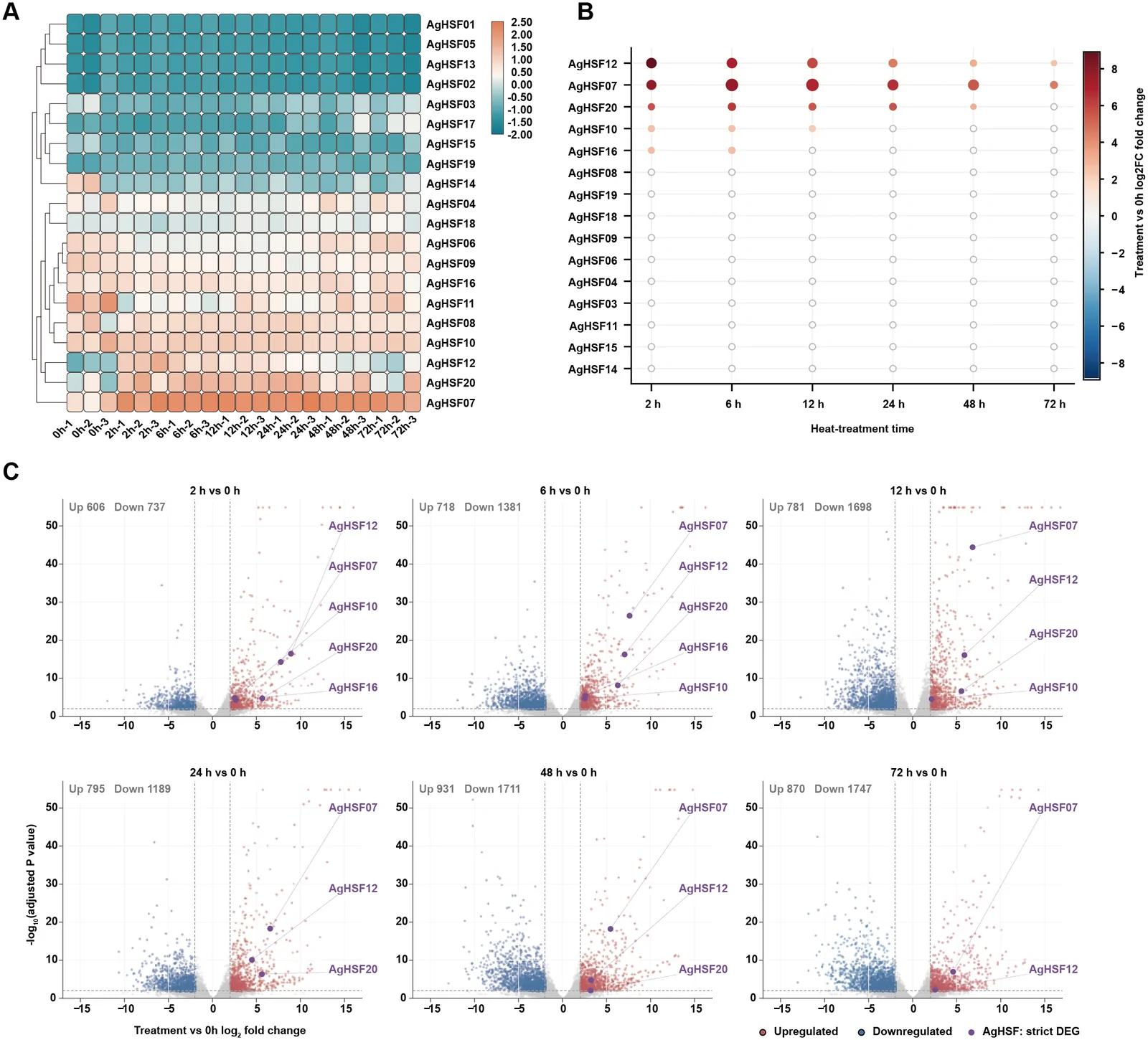
Transcriptome-based expression profiling and differential expression analysis of AgHSF genes under heat stress. **(A)** Expression profiles of the 20 AgHSF genes across seven heat-treatment time points (0, 2, 6, 12, 24, 48, and 72 h), with three biological replicates at each time point. **(B)** Temporal differential expression of AgHSF genes relative to the 0 h control. Lowly expressed genes lacking complete log_2_FC estimates were excluded from this visualization. Filled and open circles indicate genes meeting or not meeting the strict DEG criteria, respectively; color represents log_2_FC and size represents statistical significance. **(C)** Volcano plots showing transcriptome-wide differential expression at each heat-treatment time point relative to 0 h. Genes with an adjusted P value < 0.01 and |log_2_FC| ≥ 2 were defined as differentially expressed genes (DEGs).

Differential expression analysis further distinguished the magnitude and persistence of these responses (**Figures 6B, C**). Under the stringent criteria of adjusted P < 0.01 and |log_2_FC| ≥ 2, AgHSF07 and AgHSF12 were significantly differentially expressed at all six treatment time points, while AgHSF20 met the criteria at five time points. AgHSF10 and AgHSF16 showed significant responses at three and two time points, respectively. Among these genes, AgHSF07, AgHSF12, and AgHSF20 exhibited the most persistent transcriptional responses across the heat-treatment time course, whereas the responses of AgHSF10 and AgHSF16 were mainly concentrated at earlier stages.

### 3.9 Weighted gene co-expression network analysis of AgHSFs

WGCNA was performed on 24, 974 genes across 21 transcriptome samples. After expression filtering, 17, 545 genes were retained for network construction and assigned to multiple co-expression modules (**Figure 7A**). The module–time association patterns showed pronounced temporal shifts, with several modules displaying contrasting associations between the early and late stages of heat treatment.

**Figure 7:**
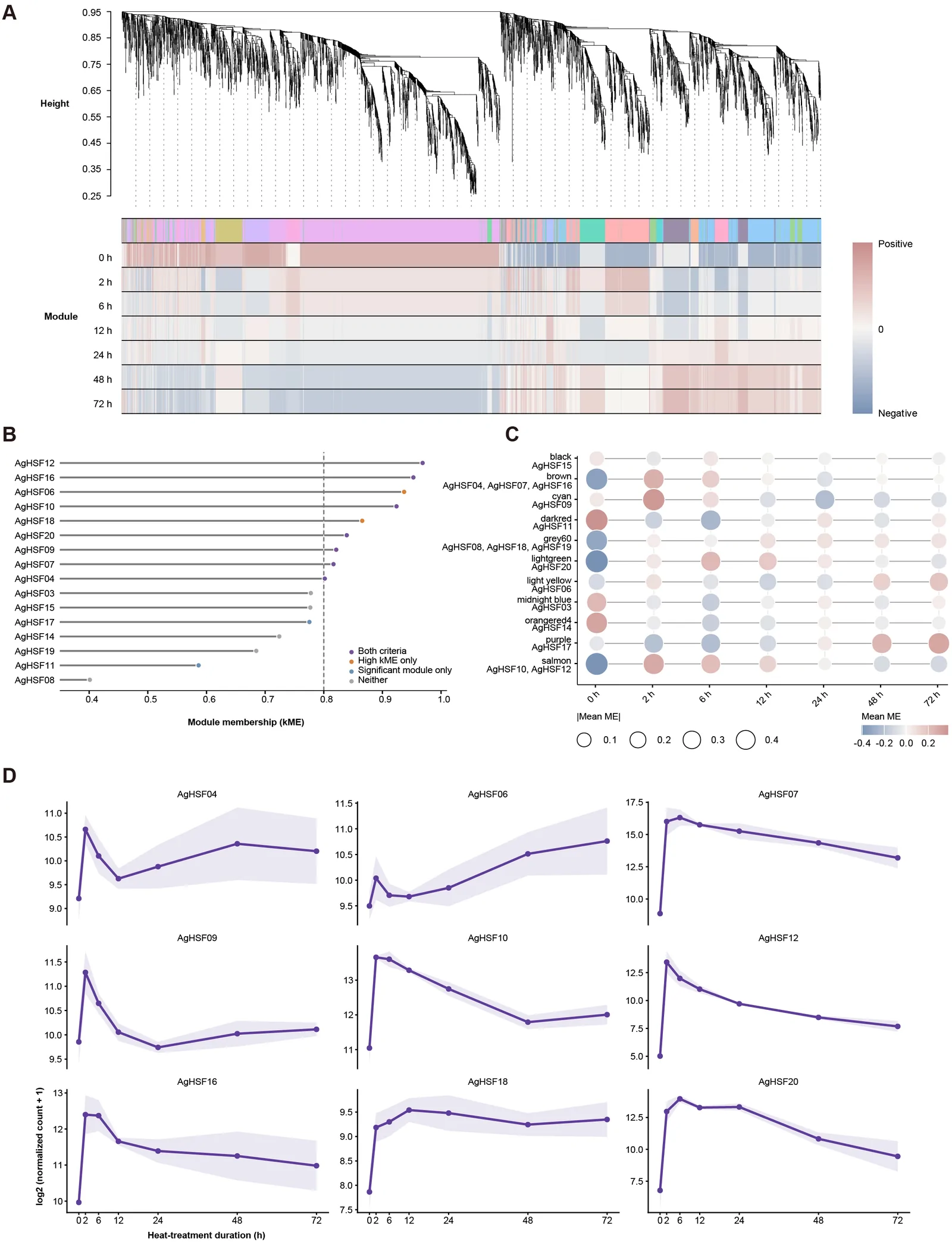
WGCNA of AgHSF-associated modules under heat stress. **(A)** Representative gene dendrogram showing WGCNA modules and their temporal associations with heat treatment. **(B)** kME distribution and module time-course significance of retained AgHSFs. **(C)** Temporal eigengene profiles of AgHSF-containing modules. Point color indicates the mean module eigengene (ME) value, and point size indicates its absolute magnitude. **(D)** Expression profiles of the nine AgHSFs with kME ≥ 0.80. Values represent mean log_2_-transformed normalized counts, with shaded areas indicating SE.

Among the AgHSFs retained in the network, nine genes showed high module membership (kME ≥ 0.80) (**Figure 7B**). Among them, seven genes, AgHSF04, AgHSF07, AgHSF09, AgHSF10, AgHSF12, AgHSF16, and AgHSF20, were located in modules with significant time-course responses (FDR < 0.05). These genes were distributed among modules with different temporal eigengene patterns (**Figure 7C**). Their expression profiles also showed distinct responses to heat treatment (**Figure 7D**). AgHSF07, AgHSF10, AgHSF12, AgHSF16, and AgHSF20 were rapidly induced at the early stages and then declined to different degrees. AgHSF09 showed a transient early response, while AgHSF04 increased early and showed a moderate increase again at later time points. Detailed differential-expression and co-expression metrics for all AgHSF genes are provided in **Supplementary Table S11**.

### 3.10 qRT-PCR validation of heat-responsive AgHSFs

To further examine the heat-responsive expression of AgHSF genes, a total of 11 AgHSF genes were selected for qRT-PCR validation under heat treatment (**Figure 8**). Distinct expression patterns were observed among these genes during the heat-treatment time course. *AgHSF04* showed relatively low expression at 0–12 h and then increased markedly at 24 h, 48 h, and 72 h. Among the analyzed genes, *AgHSF12*, *AgHSF20*, and *AgHSF07* showed particularly strong responses to heat treatment. *AgHSF12* showed low expression at 0 h, increased expression from 2 h to 12 h, and then decreased at 24–72 h. *AgHSF20* was induced after heat treatment and maintained relatively high expression from 2 h to 24 h, but showed greater variation at the later time points. *AgHSF07* exhibited a strong and sustained response, with markedly elevated expression from 2 h onward and consistently elevated relative expression throughout the subsequent treatment period. Across the 66 matched gene–time combinations, qRT-PCR and RNA-seq log_2_ fold changes were strongly positively correlated (Pearson r = 0.878, P = 4.23 × 10^-22^; Spearman ρ = 0.830, P = 7.36 × 10^-18^), providing quantitative support for the concordance between the two datasets.

**Figure 8:**
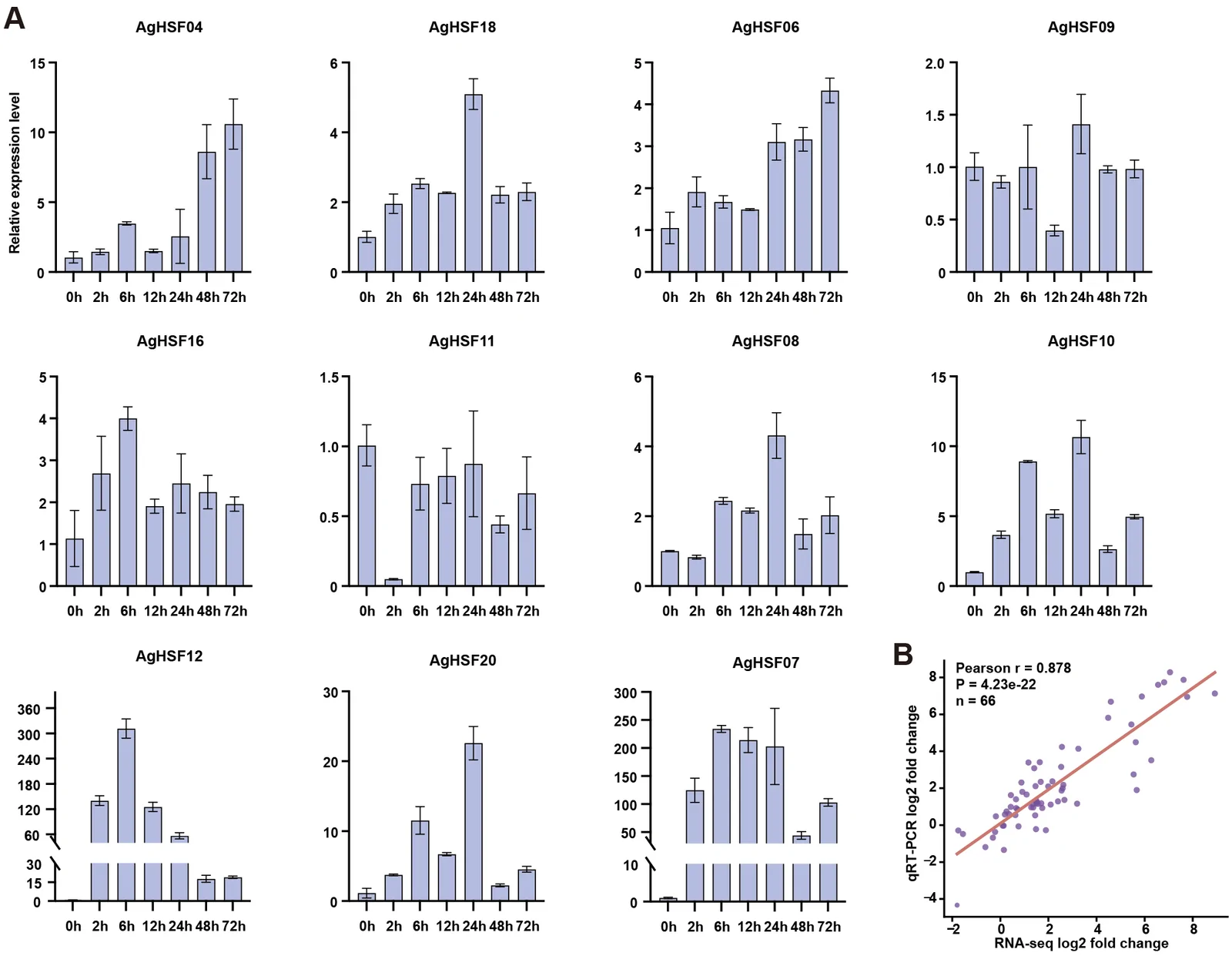
qRT-PCR validation of heat-responsive AgHSF expression. **(A)** Relative expression profiles of 11 AgHSF genes during heat treatment. Error bars represent SD of three biological replicates. **(B)** Correlation between RNA-seq and qRT-PCR log_2_ fold changes across 66 matched gene–time observations; the line represents the least-squares regression fit.

### 3.11 Subcellular localization of AgHSF07, AgHSF12, and AgHSF20

AgHSFs with strong support from heat-responsive RNA-seq profiles, differential-expression analysis, WGCNA, and qRT-PCR validation were selected for subcellular localization assays. YFP fluorescence from the empty-vector control was broadly distributed in *Nicotiana benthamiana* leaf epidermal cells (**Figure 9**). The YFP signals of AgHSF07 and AgHSF12 were predominantly concentrated in the nuclei, indicating that both proteins were mainly localized to the nucleus. In contrast, the AgHSF20 signal was mainly distributed at the cell periphery, with no obvious nuclear enrichment.

**Figure 9:**
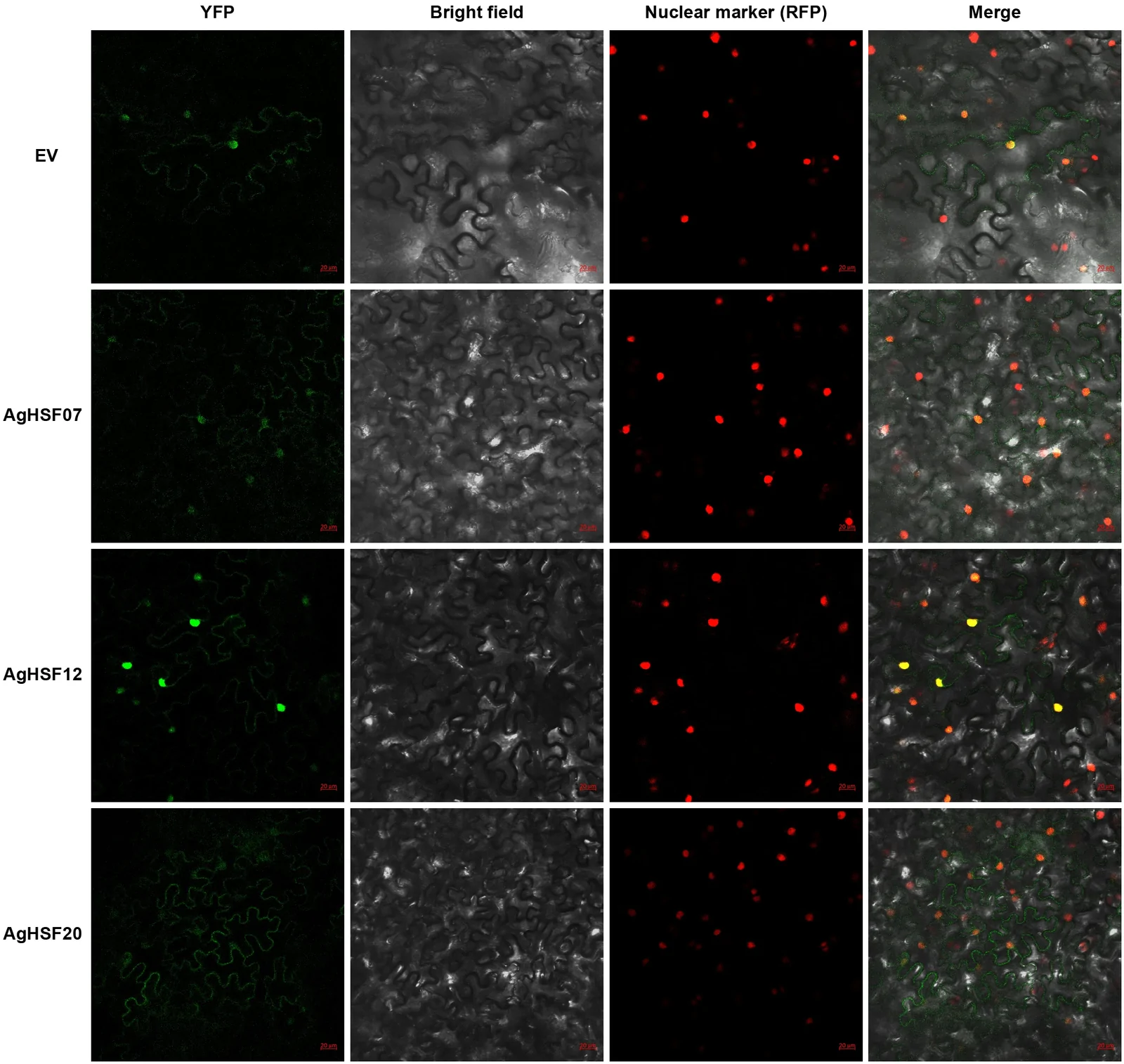
Subcellular localization of AgHSF07, AgHSF12, and AgHSF20 in *Nicotiana benthamiana* leaf epidermal cells. Columns show YFP fluorescence, bright-field images, nuclear marker (RFP), and merged images. Scale bars = 20 μm.

### 3.12 Transcriptional activation and yeast two-hybrid assays of selected AgHSFs with HSE analysis of candidate partner genes

GAL4-based yeast assays revealed distinct transcriptional activation properties among the three AgHSF proteins (**Supplementary Figure S2**). BD-AgHSF07 and BD-AgHSF12 showed strong autoactivation, with AgHSF07 displaying stronger activity, whereas BD-AgHSF20 showed no detectable autoactivation. These results identified intrinsic transactivation as a major regulatory property of AgHSF07 and AgHSF12 in yeast.

Candidate interaction partners were subsequently selected from the STRING-derived protein association network (**Supplementary Table S9**). HSP70–4 showed strong predicted associations with AgHSF07 and AgHSF20, with combined scores of 0.862 and 0.916, respectively, whereas HSP90–1 showed the strongest predicted association with AgHSF12, with a combined score of 0.987 (**Supplementary Table S9**). Based on sequence similarity and expression-profile concordance, KAK1264716, KAK1261613, and KAK1264717 were selected as candidate partners of AgHSF07, AgHSF12, and AgHSF20, respectively (**Supplementary Table S12**). The candidate partner BD fusions showed no detectable autoactivation. The tested AgHSF–partner combinations did not activate the selective markers, indicating that direct interactions were not detected under the conditions tested.

HSEs with three contiguous pentameric units were identified in the 2-kb upstream sequences of KAK1264716 and KAK1261613, the candidate partner genes of AgHSF07 and AgHSF12, respectively. These HSEs showed the NGAAN–NTTCN–NGAAN configuration (**Supplementary Table S13**). No such HSE was detected in the 2-kb upstream sequence of KAK1264717, the candidate partner gene of AgHSF20.

## 4 Discussion

In this study, the HSF family comprised 39 members in *A. calamus* and 20 in *A. gramineus*, and all *Acorus* HSFs were assigned to the three canonical subfamilies, HSFA, HSFB, and HSFC (**Figure 1**). This conserved three-group pattern is consistent with reports from many other angiosperms, although the total family size varies substantially among species (Deng et al., 2024; Kang et al., 2025; Zhao et al., 2026). In our dataset, HSFA and HSFB accounted for most of the family members, whereas HSFC contained only three genes in total. Previous studies likewise describe HSFA as the largest subfamily, whereas HSFC generally contains fewer and less-characterized members than HSFA or HSFB (Li et al., 2025). HSFA1 and HSFA2 act as major positive regulators of heat responses, with HSFA1 functioning upstream and HSFA2 contributing to sustained and acquired thermotolerance (Liu and Charng, 2013; Fragkostefanakis et al., 2016), whereas HSFB proteins mainly act as transcriptional repressors or co-regulators depending on the genetic background and stress context (Ikeda et al., 2011; Guo et al., 2016). The difference in ploidy between tetraploid *A. calamus* and diploid *A. gramineus* may partly account for the larger HSF complement in *A. calamus*. In many plant lineages, increases in gene family size have been linked to polyploidy, whole-genome duplication, and large segmental duplication events (Zhu et al., 2017; Xie et al., 2023; Zhu et al., 2025; Zhao et al., 2026). However, genome duplication does not always produce a proportional increase in family size, because duplicated genes can subsequently be lost or retained at different rates (Zhu et al., 2017; Yu et al., 2022). The present results indicate that *Acorus* HSFs retain the conserved structural framework characteristic of plant heat shock transcription factors, while the larger family size in tetraploid *A. calamus* reflects species-specific evolutionary history relative to diploid *A. gramineus*.

All AcHSF and AgHSF proteins contained the HSF domain and motif 1, whereas the remaining motifs varied among branches and subfamilies (**Figure 2**). Closely related gene pairs or clusters showed similar motif arrangements, while the three HSFC members displayed a compact and distinct motif pattern. This pattern is consistent with previous studies, in which proteins within the same subfamily usually display similar motif composition and domain organization, whereas different subfamilies are more clearly separated at the structural level (Zhu et al., 2025; Hu et al., 2026). These results indicate that plant HSFs share a conserved DNA-binding core but differ in additional structural features associated with subfamily identity and regulatory specialization.

The interspecific collinearity analysis showed that several AgHSFs corresponded to more than one AcHSF, indicating that HSF relationships between the two species are not strictly one-to-one (**Figure 3**). Segmental duplication is recognized as a major mechanism of HSF family expansion and is often followed by differential gene retention (Deng et al., 2024; Kang et al., 2025). The HSF relationship between the two species is more complex than a simple two-fold pattern, suggesting differential loss and retention of duplicated genes after polyploidization (Yu et al., 2022; Kong et al., 2023). Overall, the present HSF distribution likely reflects the combined effects of shared ancestry, polyploidization, gene loss, and differential retention.

Polyploidization can generate asymmetry in both gene retention and gene expression, but these two processes do not necessarily follow the same pattern (Liang and Schnable, 2018). Our results showed that the AcHSF family did not exhibit the subgenome-biased gene retention reported at the genome-wide level in *A. calamus*. However, ten of the 14 retained AcHSF homeolog pairs showed expression bias in at least one tissue, and B-biased expression occurred twice as often as A-biased expression (**Supplementary Figure S1**). This tendency agrees with the genome-wide expression pattern reported for *A. calamus*, in which more homeologous gene pairs showed higher expression from subgenome B than from subgenome A (Ma et al., 2023). A similar pattern has been reported in synthetic *Brassica napus*, where subgenome-biased expression was detected even in regions with balanced homeolog dosage (Bird et al., 2021). Such expression asymmetry can arise soon after genome merger and may become stronger during subsequent evolution, reflecting regulatory changes between retained homeologs (Yoo et al., 2013). Therefore, although the AcHSF family retained a nearly balanced number of genes from the two subgenomes, the retained homeologs showed clear expression divergence with an overall tendency toward greater contribution from subgenome B.

Promoter analysis showed that AcHSF and AgHSF genes contain diverse regulatory elements (**Figure 4**). The frequent occurrence of ABRE suggests a link between HSF regulation and ABA signaling under abiotic stress, consistent with the function of *Arabidopsis* HSFA6b (Huang et al., 2016). The widespread light-responsive elements also indicate possible regulation by light-related signals (Xie et al., 2023; Zhao et al., 2026). These observations support the view that *Acorus* HSF promoter architecture integrates light cues, hormone signaling, and abiotic stress responses.

The predicted interaction network and GO enrichment results together support the involvement of AgHSFs in canonical heat-response pathways (**Figure 5**). The predicted partners of several AgHSFs were mainly HSP family members and other heat-related proteins, indicating that AgHSFs may participate in HSF–HSP-associated heat responses. A similar pattern has been reported in previous studies, in which HSPs function as core chaperones downstream of HSFs (Schramm et al., 2008; Meiri and Breiman, 2009; Hsu et al., 2010). GO enrichment in transcriptional regulation and heat-response terms further supports the involvement of AgHSFs in plant heat-stress regulation (**Figure 5B**).

Transcriptome profiling, DEG analysis, WGCNA, and qRT-PCR showed that several AgHSFs responded strongly to heat treatment (**Figures 6**–**8**; **Supplementary Table S11**). The family members differed not only in response magnitude but also in response duration and co-expression context. Similar differences among HSF family members have been reported in other plants, where only a subset of HSF genes showed strong stress-induced expression (Deng et al., 2024; Kang et al., 2025). This interpretation is consistent with the established functional diversification of plant HSF families (Guo et al., 2016; Ohama et al., 2017). Therefore, AgHSF members may act at different phases or regulatory layers of the heat response, with *AgHSF07*, *AgHSF12*, and *AgHSF20* representing the strongest candidates for subsequent functional analysis.

Plant HSF activity is closely regulated by molecular chaperone systems involving HSP70 and HSP90 (Zhao et al., 2021b; Toribio et al., 2024), while many HSFAs function as transcriptional activators and central regulators of plant heat responses (Wu et al., 2022; Li et al., 2023; Huang et al., 2023). In this study, the GAL4-based yeast assay was used to assess the intrinsic transcriptional activation of selected AgHSFs and their possible interactions with predicted partners. AgHSF07 and AgHSF12 showed strong bait autoactivation, supporting their transcriptional activation potential in yeast (**Supplementary Figure S2**). Previous studies using GAL4-based yeast assays have shown that transcriptionally active HSFAs often function as major positive regulators of plant heat responses. In wheat, TaHsfA1 showed clear transcriptional activation activity in yeast, and loss-of-function mutants exhibited impaired induction of heat-responsive genes and compromised both basal and acquired thermotolerance, indicating an important upstream role in heat-stress regulation (Wang et al., 2023). Similarly, ZmHsf17 and OsHSFA4d showed transcriptional activation activity in GAL4-based yeast assays and were subsequently demonstrated to positively regulate thermotolerance (Fang et al., 2025; Zhang et al., 2025). Our structural analysis showed that AgHSF07 and AgHSF12 both contained predicted NLS, NES, and AHA motifs. NLS and NES are involved in the nuclear import and export of HSF proteins, while AHA motifs are important for the transcriptional activation of class A HSFs (Lyck et al., 1997; Kotak et al., 2004; Guo et al., 2016). These structural features are consistent with the predominant nuclear localization and strong autoactivation of AgHSF07 and AgHSF12. Although no direct interactions were detected with the selected partners, HSEs were identified in the upstream regions of the candidate partner genes of AgHSF07 and AgHSF12. Previous studies have shown that HSFs can bind HSEs in the promoters of downstream genes and activate their transcription, including several HSP genes (Xue et al., 2014; Albertos et al., 2022). These results suggest that the candidate genes may be transcriptional targets of AgHSF07 and AgHSF12. Therefore, together with their HSFA classification, structural features, predominant nuclear localization, and strong heat-responsive expression, these findings support AgHSF07 and AgHSF12 as candidate transcriptional activators and putative regulators of heat responses in *Acorus*.

In contrast, AgHSF20 showed mainly cytoplasmic fluorescence (**Figure 9**). Previous studies have also reported cytoplasmic localization for some plant HSFs, such as tomato HsfA2 and *Arabidopsis* HsfA6a (Scharf et al., 1998; Hwang et al., 2014). Our analysis showed that AgHSF20 belongs to the HSFB2 subclass, but no canonical nuclear localization signal (NLS) was predicted (**Supplementary Table S4**), which is consistent with previous evidence that the nuclear localization of *Arabidopsis* HsfB2b depends on its NLS, as deletion of the NLS-containing region results in cytoplasmic localization (Zhu et al., 2012). Therefore, the cytoplasmic localization of AgHSF20 may be partly related to weak nuclear import. Overall, this work presents the first comprehensive analysis of heat shock transcription factors in *Acorus*, revealing their evolutionary, genomic, and heat-responsive expression features and providing a foundation for further investigation of HSF-mediated heat-response mechanisms. Future studies using Dual-LUC, transient overexpression, VIGS, and stable transformation in Acorus or heterologous systems will further examine the transcriptional regulatory activities of the selected AgHSFs and their contributions to thermotolerance.

## 5 Conclusion

This work presents the first integrated analysis of HSFs in *Acorus*. The tetraploid *A. calamus* genome contained 39 HSF genes, compared with 20 in diploid *A. gramineus*. Phylogenetic, structural, and syntenic analyses revealed overall conservation of the HSF family and indicated that polyploidization and differential gene retention contributed to HSF family expansion in *A. calamus*. Although the 39 AcHSFs were nearly equally distributed between the A and B subgenomes, the retained homeolog pairs showed an overall tendency toward greater expression from the B subgenome, indicating divergence in their regulatory contributions after allopolyploidization. The combined expression, differential-expression, co-expression, and qRT-PCR evidence further demonstrates substantial temporal and regulatory differentiation among AgHSF members, with AgHSF07, AgHSF12, and AgHSF20 showing particularly prominent responses to heat treatment. Subcellular localization and GAL4-based yeast assays further showed that AgHSF07 and AgHSF12 were predominantly nuclear proteins with intrinsic transcriptional activation activity, supporting their roles as candidate regulators of heat responses in *Acorus*. Overall, these findings advance our understanding of HSF evolution and heat-response regulation in *Acorus* and provide candidate genetic resources for future studies aimed at improving thermotolerance.

## Data Availability

The raw reads are available in the China National GeneBank (CNGB) Nucleotide Sequence Archive (CNSA) under accession number CNP0009295.
