# Supplementary material for: Integrative characterization of the heat shock transcription factors in Acorus: evolution and heat-responsive regulatory patterns
Source: Front Plant Sci. 2026 Oct 7;17:1974158. doi: 10.3389/fpls.2026.1974158 (PMC13682968; doi:10.3389/fpls.2026.1974158)
Supplement: Supplementary file 1 [file DataSheet1.pdf]

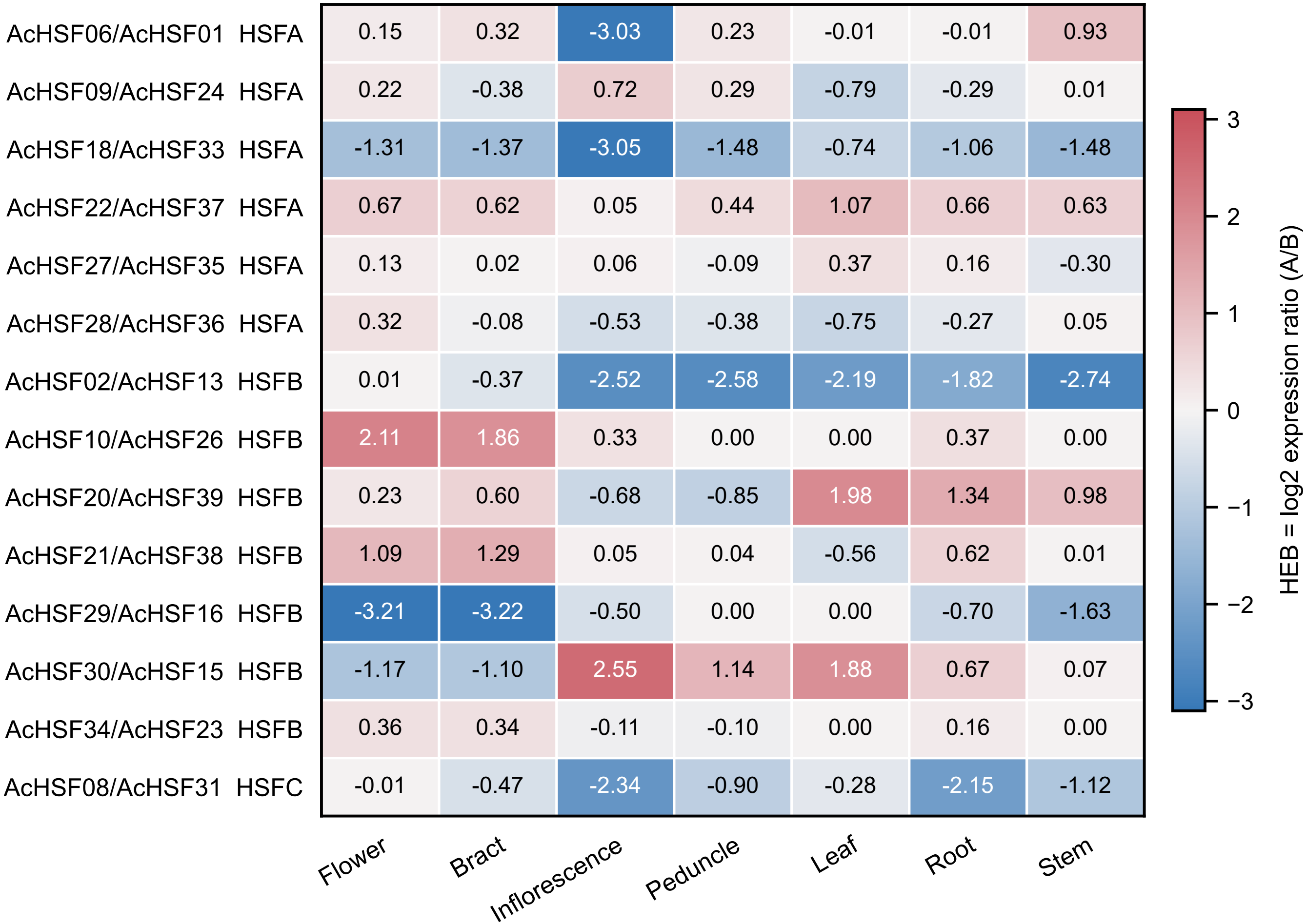
AcHSF06/AcHSF01 HSFA
0.15 0.32 -3.03 0.23 -0.01 -0.01 0.93
AcHSF09/AcHSF24 HSFA
0.22 -0.38 0.72 0.29 -0.79 -0.29 0.01
AcHSF18/AcHSF33 HSFA
-1.31 -1.37 -3.05 -1.48 -0.74 -1.06 -1.48
AcHSF22/AcHSF37 HSFA
0.67 0.62 0.05 0.44 1.07 0.66 0.63
AcHSF27/AcHSF35 HSFA
0.13 0.02 0.06 -0.09 0.37 0.16 -0.30
AcHSF28/AcHSF36 HSFA
0.32 -0.08 -0.53 -0.38 -0.75 -0.27 0.05
AcHSF02/AcHSF13 HSFB
0.01 -0.37 -2.52 -2.58 -2.19 -1.82 -2.74
AcHSF10/AcHSF26 HSFB
2.11 1.86 0.33 0.00 0.00 0.37 0.00
AcHSF20/AcHSF39 HSFB
0.23 0.60 -0.68 -0.85 1.98 1.34 0.98
AcHSF21/AcHSF38 HSFB
1.09 1.29 0.05 0.04 -0.56 0.62 0.01
AcHSF29/AcHSF16 HSFB
-3.21 -3.22 -0.50 0.00 0.00 -0.70 -1.63
AcHSF30/AcHSF15 HSFB
-1.17 -1.10 2.55 1.14 1.88 0.67 0.07
AcHSF34/AcHSF23 HSFB
0.36 0.34 -0.11 -0.10 0.00 0.16 0.00
AcHSF08/AcHSF31 HSFC
-0.01 -0.47 -2.34 -0.90 -0.28 -2.15 -1.12
Flower
Bract
Inflorescence
Peduncle
Leaf
Root
Stem
3
2
1
0
−1
−2
−3
HEB = log2 expression ratio (A/B)
