# Supplementary material for: Integrative characterization of the heat shock transcription factors in Acorus: evolution and heat-responsive regulatory patterns
Source: Front Plant Sci. 2026 Oct 7;17:1974158. doi: 10.3389/fpls.2026.1974158 (PMC13682968; doi:10.3389/fpls.2026.1974158)
Supplement: Supplementary file 2 [file DataSheet2.pdf]

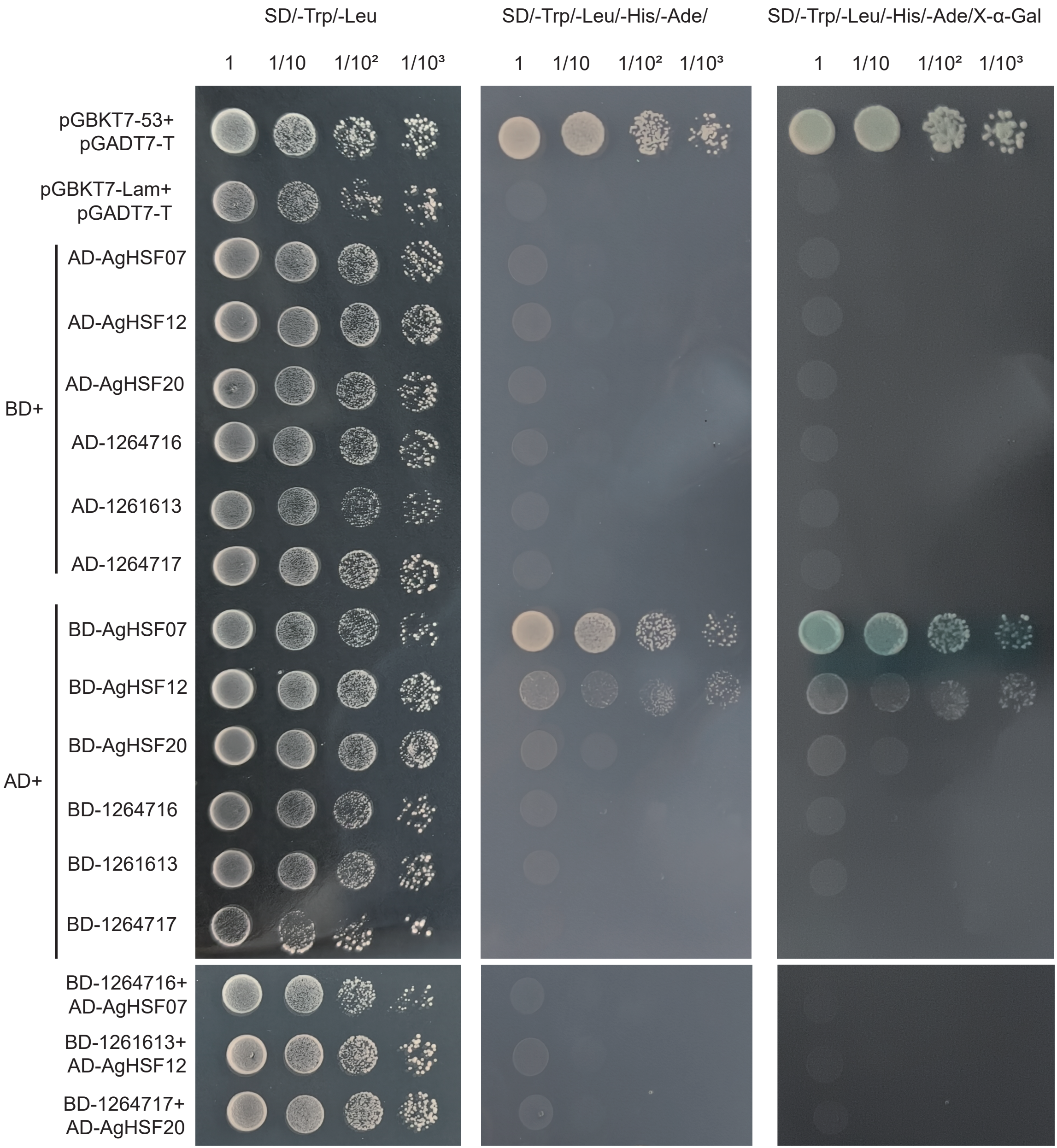
SD/-Trp/-Leu
SD/-Trp/-Leu/-His/-Ade/
SD/-Trp/-Leu/-His/-Ade/X-α-Gal
1
1/10
1/10²
1/10³
pGBKT7-53+ pGADT7-T
pGBKT7-Lam+ pGADT7-T
BD+
AD-AgHSF07
AD-AgHSF12
AD-AgHSF20
AD-1264716
AD-1261613
AD-1264717
AD+
BD-AgHSF07
BD-AgHSF12
BD-AgHSF20
BD-1264716
BD-1261613
BD-1264717
BD-1264716+ AD-AgHSF07
BD-1261613+ AD-AgHSF12
BD-1264717+ AD-AgHSF20
